# Working memory deficits in schizophrenia are associated with the rs34884856 variant and expression levels of the *NR4A2* gene in a sample Mexican population: a case control study

**DOI:** 10.1186/s12888-021-03081-w

**Published:** 2021-02-09

**Authors:** Elizabeth Ruiz-Sánchez, Janet Jiménez-Genchi, Yessica M. Alcántara-Flores, Carlos J. Castañeda-González, Carlos L. Aviña-Cervantes, Petra Yescas, María del Socorro González-Valadez, Nancy Martínez-Rodríguez, Antonio Ríos-Ortiz, Martha González-González, María E. López-Navarro, Patricia Rojas

**Affiliations:** 1 Laboratory of Neurotoxicology, Instituto Nacional de Neurología y Neurocirugía, “Manuel Velasco Suárez”, SS, Av. Insurgentes Sur No. 3877, Col. La Fama, C.P. 14269 Mexico City, Mexico; 2 Research Unit, Hospital Psiquiátrico Fray Bernardino Álvarez, Mexico City, Mexico; 3 General Direction, Hospital Psiquiátrico Fray Bernardino Álvarez, Mexico City, Mexico; 4 Department of Psychiatry, Instituto Nacional de Neurología y Neurocirugía, “Manuel Velasco Suárez”, SS, Av. Insurgentes Sur No. 3877, Col. La Fama, C.P. 14269 Mexico City, Mexico; 5 Department of Genetics, Instituto Nacional de Neurología y Neurocirugía, “Manuel Velasco Suárez”, SS, Av. Insurgentes Sur No. 3877, Col. La Fama, C.P. 14269 Mexico City, Mexico; 6 Health Care Division, Hospital Psiquiátrico Fray Bernardino Álvarez, Mexico City, Mexico; 7 Epidemiology, Endocrinology & Nutrition Research Unit, Hospital Infantil de México “Federico Gómez”, Mexico City, Mexico; 8 Unit of Cognition and Behavior, Instituto Nacional de Neurología y Neurocirugía, “Manuel Velasco Suárez”, SS, Av. Insurgentes Sur No. 3877, Col. La Fama, C.P. 14269 Mexico City, Mexico

**Keywords:** Schizophrenia, *NR4A2* genetic variants, *NR4A2* mRNA levels, Cognition, Endophenotype, Working memory

## Abstract

**Background:**

Cognitive functions represent useful endophenotypes to identify the association between genetic variants and schizophrenia. In this sense, the *NR4A2* gene has been implicated in schizophrenia and cognition in different animal models and clinical trials. We hypothesized that the *NR4A2* gene is associated with working memory performance in schizophrenia. This study aimed to analyze two variants and the expression levels of the *NR4A2* gene with susceptibility to schizophrenia, as well as to evaluate whether possession of *NR4A2* variants influence the possible correlation between gene expression and working memory performance in schizophrenia.

**Methods:**

The current study included 187 schizophrenia patients and 227 controls genotyped for two of the most studied *NR4A2* genetic variants in neurological and neuropsychiatric diseases. Genotyping was performed using High Resolution Melt and sequencing techniques. In addition, mRNA expression of *NR4A2* was performed in peripheral mononuclear cells of 112 patients and 118 controls. A group of these participants, 54 patients and 87 controls, performed the working memory index of the WAIS III test.

**Results:**

Both genotypic frequencies of the two variants and expression levels of the *NR4A2* gene showed no significant difference when in patients versus controls. However, patients homozygous for the rs34884856 promoter variant showed a positive correlation between expression levels and auditory working memory.

**Conclusions:**

Our finding suggested that changes in expression levels of the *NR4A2* gene could be associated with working memory in schizophrenia depending on patients’ genotype in a sample from a Mexican population.

**Supplementary Information:**

The online version contains supplementary material available at 10.1186/s12888-021-03081-w.

## Background

Schizophrenia is a heterogeneous, severe, and disabling mental illness that affects 1% of the world’s population and is caused by interactions between environmental and genetic factors [1]. It has also considered a neurodevelopmental disorder with alterations in different neurotransmitters [2], and dopamine plays an essential role in this disease.

It is recognized by diverse symptoms, with cognitive deficits being a core feature of schizophrenia [3], and which are present in up to 98% of patients [4]. Several studies show that schizophrenia and cognitive dysfunction manifest considerable heritability between 70 to 90% and 24 to 55%, respectively [5, 6]. In particular, cognitive functions, such as working memory, have been proposed as endophenotypes related to schizophrenia [7]. An endophenotype is a subtype of biomarker, a quantitative biological trait that is thought to be related to genetic vulnerability and disease onset [8], and is characterized by being intermediate, measurable, heritable and independent traits of the disease status (present both in the prodromal stage and in patients with remission) [9].

Different genes involved in the neurodevelopment of dopaminergic neurons can be considered candidate susceptibility genes for schizophrenia and cognitive deficits [10, 11], for instance, the gene that codes for the nuclear receptor subfamily 4, group A (NR4A2). This transcription factor regulates the expression of genes involved in the development, survival, and phenotype of dopaminergic neurons [12, 13]. The *NR4A2* gene (also known as *Nurr1*) has been related to schizophrenia etiology and cognitive function [10, 14–18]. Notably, in relation to this gene, Nurr1 heterozygous (+/−) mice show cognitive impairment and pharmacological responses consistent with a model for schizophrenia [19, 20]. This correlation between *NR4A2* deficiency and cognitive skills has been found in other animal models for Alzheimer’s disease and attention-deficit hyperactivity disorder [18, 21] as well as its particular role in memory tasks as reported in preclinical studies [17, 22–25].

On the other hand, different clinical studies have analyzed genetic variants and gene expression levels, in peripheral blood mononuclear cells (PBMC), both as potential biomarkers for central nervous system disorders [26, 27]. In this way, several genetic variants of the *NR4A2* gene have been analyzed, giving rise to non-reproducible results in different populations [28–33]. The two genetic variants analyzed in the present study have been associated with other neurological diseases, psychiatric disorders, and addiction, which are also related with dysfunction of the dopaminergic system. In particular, the rs35479735 intronic 6 variant has been associated with Parkinson’s disease in Asian, Caucasian, and Mexican populations [34–36]. In addition, the rs34884856 promoter variant has been associated with alcohol dependence in people with Mexican ancestry [37].

The association between the *NR4A2* gene and cognitive deficit in schizophrenia has been previously reported in a Caucasian population [32]. In that study, Ancin et al. 2013 [32] have identified an association between *NR4A2* variants and cognition (sustained attention) in schizophrenia patients. In addition, preclinical studies have been shown the importance of the *NR4A2* gene in cognition. Therefore, to study this association is of importance in psychiatry field, especially in schizophrenia.

Furthermore, decreased expression of the *NR4A2* gene was found in the dorsolateral prefrontal cortex (DLPFC) of patients with schizophrenia [14, 38, 39]. However, the relationship of *NR4A2* expression in PBMC with gene variants in schizophrenia remains unclear. Likewise, the expression of genes related to inflammation, metabolism, and neuroprotection from PBMC have linked to alterations in the activity of brain regions [40, 41].

To our knowledge, there is a lack of studies on the relation of the *NR4A2* gene with working memory deficits in schizophrenia, as well as the need to prioritize diversity in human genomics research [42] in the field of Psychiatry. Therefore, we investigate the association between the *NR4A2* gene with working memory deficit in schizophrenia in the Mexican population, which is under-represented in human genomic research.

We hypothesized that the *NR4A2* gene is associated with working memory performance in schizophrenia in a sample Mexican population. Therefore, this study aimed to analyze the genotype effect of two genetic variants (rs34884856 promoter variant and rs35479735 intronic 6 variant), and the expression levels of the *NR4A2* and their interactions on performance of working memory ability in schizophrenia.

The association analysis of genetic variants and levels of expression with schizophrenia was first evaluated, followed by the relationship of expression with variants, and finally the relationship of cognition with expression and variants. Thus, the interactions analyzed of working memory were diagnosis-genotype (genetic variants), genotype-expression, and diagnosis-genotype-expression.

## Methods

### Participants

This study included 187 (111 males and 76 females) patients diagnosed with schizophrenia at the Instituto Nacional de Neurología “Manuel Velasco Suárez” and Hospital Psiquiátrico Fray Bernardino Álvarez from March 2012 to December 2017. Inclusion criteria: patients confirmed by two psychiatrists, following Diagnostic and Statistical Manual of Mental Disorders Fourth Edition (DSM-IV) criteria [43] and the Composite International Diagnostic Interview (CIDI) Version 21 [44], and with at least 1 year of evolution. Exclusion criteria included: patients with comorbidities due to toxic substance use (except nicotine) during 3 months prior to recruitment, or other serious organic or neurological diseases.

The control volunteers consisted of 227 unrelated participants (127 males and 100 females) and recruited from March 2011 to December 2017 through announcement from population of Mexico City’s metropolitan area. This group was screened by psychiatrists and completed the Mini-International Neuropsychiatric Interview (MINI) [45] to rule out any personal history of neuropsychiatric disorder. After clinical evaluation the control group was matched for age and sex with the group of cases.

Control volunteers were without history of substance abuse (except nicotine), and with no family history from of schizophrenia, or other neurological, or psychiatric disorders.

**Fig. 1:**
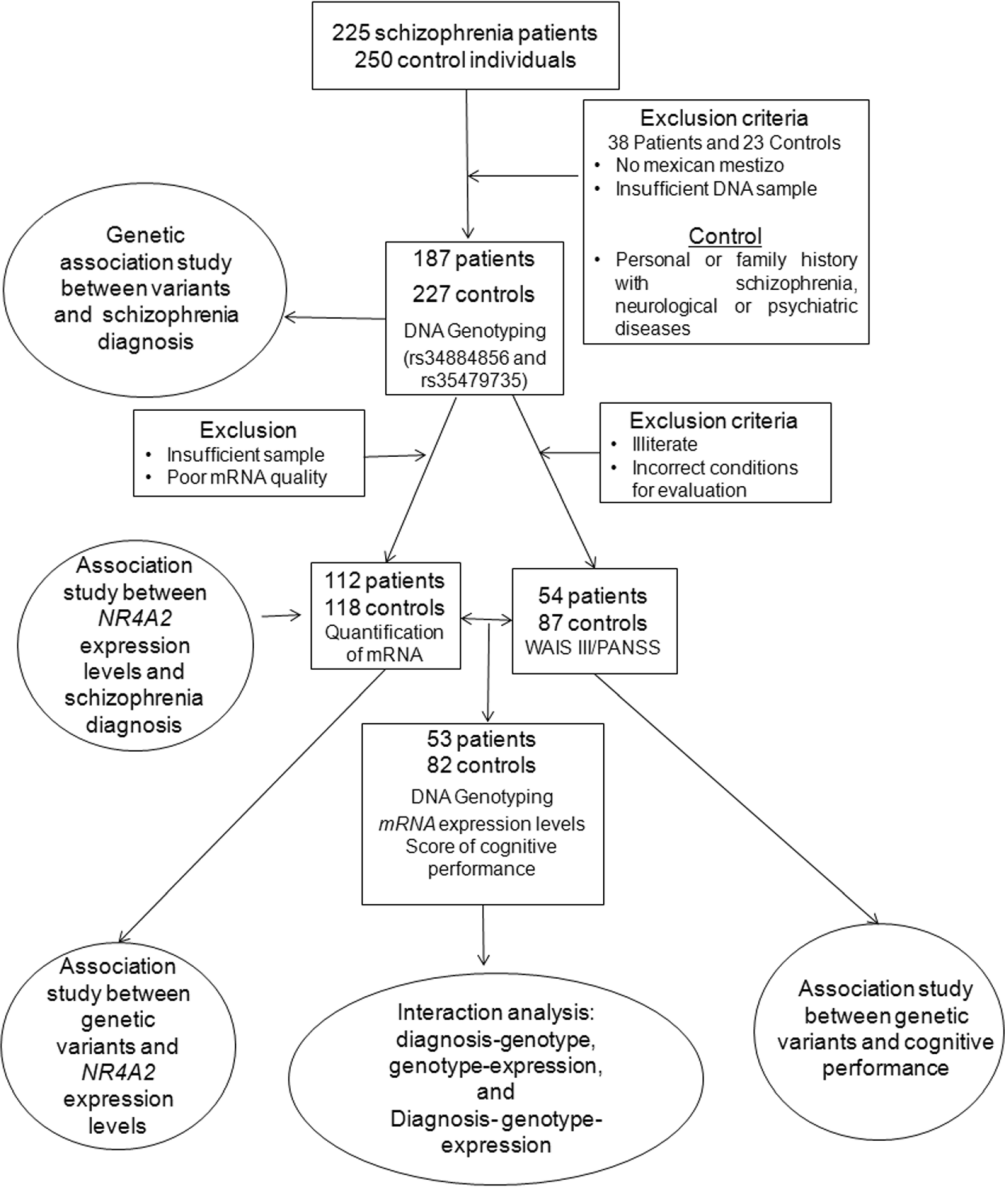
Study flow-chart

Both the patients and control group met the criteria of Mexican mestizo [46]. The analysis did not include ancestral-informative markers (AIMs). However, standard criteria were used to define the Mexican mestizo, and to keep the effects of population stratification to a minimum. According to the National Institute of Anthropology and History of Mexico, Mestizos are defined as individuals born in Mexico, having a Spanish-derived last name, with family antecedents of Mexican ancestors back at least to the third generation [47]. The study was performed in accordance with principles of the 1964 Helsinki Declaration and its later amendments. The institutional ethics committee of the Instituto Nacional de Neurología y Neurocirugía, “Manuel Velasco Suárez” and Hospital Psiquiátrico Fray Bernardino Álvarez approved the study protocol, and written informed consent was obtained from all individual participants included in the study. The experimental design is show in Fig. 1.

The 187 schizophrenia patients and 227 controls were genotyped for two of the most studied *NR4A2* genetic variants in neurological and neuropsychiatric diseases (rs34884856 promoter variant and rs35479735 intronic 6 variant) (see below; Molecular analysis).

### Clinical and working memory evaluation

The working memory function was evaluated in a group of patients and controls (54 patients with paranoid schizophrenia and 87 controls) of the total of participants using the Wechsler Adult Intelligence Scale (WAIS-III) standardized for the Mexican population [48]. This scale was conducted in literate patients and controls. In addition, it was also confirmed that they were not undergoing any treatment associated with drowsiness (benzodiazepine) and that they met the conditions required to obtain a correct cognitive evaluation. The severity of the schizophrenia symptoms was evaluated in the same patients by means of the Positive and Negative Syndrome Scale (PANSS) [49]. Clinical evaluation and cognitive tests (WAIS-III) were carried out between 2012 and 2017 by psychiatry and psychology experts, previously trained in the application of both tests. Because the present study aimed to evaluate working memory in schizophrenia, the subtests that evaluate this task are part of the WAIS-III. The experimental design is shown in Fig. 1.

WAIS is one of the most widely used tests to assess general intellectual ability in adults 16 years of age and older [50, 51]. From its original [52] development this test has been revised on several occasions. The WAIS-III is the fifth version of the intelligence scale and introduced four index scores derived from factor analyses of 14 subtests. The four index scores, Verbal Comprehension (VC), Perceptual Organization (PO), Working Memory (WM), and Processing Speed (PS) divide abilities into more discrete units of functioning compared to the traditional IQ scores [53].

The Working Memory Index (WMI) is commonly used to evaluate working memory ability. The WAIS-III WMI is derived from the performance on the Digit Span (DS), Arithmetic, and Letter-Number Sequencing subtests.

To evaluate the association between the *NR4A2* gene and working memory in schizophrenia, WMI and its three subtests (DS, Arithmetic, and Letter-Number Sequencing) that compose it were analyzed with genetic variants and the expression levels of the *NR4A2* gene. The Backward Digit Span (BDS) task, aural evaluation that is part of the DS subtest was considered for analysis. The WMI score was obtained from WAIS-III.

The DS subtest is composed of both a forward and a backward recitation condition. On the digits forward part of the subtest, the individual is verbally presented with a string of numbers and asked to repeat back the numbers in order immediately after stimuli presentation. In the digits backward condition, the individual is verbally instructed to repeat back the presented string of numbers in reverse order. The DS score combines the total number of digit strings correctly repeated in both conditions.

### Molecular analysis

#### DNA and RNA extraction

The PBMC obtained from the patients were stored in stabilization solution (RNAlater, Invitrogen) at − 20 °C until processing (DNA and RNA) to prevent the degradation of genetic material. The concentration and purity of the RNA and DNA samples were measured by spectrophotometric analysis. The integrity of the samples (DNA and RNA) was determined by horizontal electrophoresis in 1.5% agarose / TAE 1X gels at 100 V / 30 min [54]. RNA and DNA samples with a concentration greater than 50 ng/μl, a 260/280 ratio between 1.8 to 2 and with an electrophoretic pattern for high molecular weight DNA and 18 s and 28 s ribosomal RNA were chosen for the genotyping study and of genetic expression.

#### Genotyping

Genomic DNA was extracted from PBMC using the standard salting out procedure [55]. Two single nucleotide variants (SNV) of *NR4A2*, rs35479735 in intron 6 and rs34884856 in the promoter, were genotyped in patients and the control group. Genotyping was performed using High Resolution Melt (HRM) and sequencing techniques. The oligonucleotide design and conditions of both genotyping methods have been previously described [36]. HRM analysis was performed using the Rotor-Gene 6000 instrument (Corbett Research Pty Ltd., Sydney, Australia). All analyzes were performed in triplicates.

#### mRNA expression levels of *NR4A2*

*NR4A2* expression levels were evaluated in 112 patients and 118 control individuals. *NR4A2* mRNA extraction was performed in PBMC by the organic technique using the TRIzol reagent, following the manufacturer’s instructions. Quantification of the expression levels of the *NR4A2* gene was carried out by qRT-PCR, as previously reported [36].

GAPDH was used as an internal reference gene to normalize target gene transcript levels. The quantitative PCR reactions were performed using predesigned primer and probes from Applied Biosystems (Foster City, CA, USA). The relative fold changes were determined by the method of 2^−ΔΔCt^ as described previously [56]. The analysis was carried out in triplicate.

### Statistical analysis

The study population is described using means and standard deviations or frequencies and percentages. Mann-Whitney *U* tests for non-parametric data, Student t tests for continuous parametric data, and chi-square tests for categorical parameters were used. The Hardy-Weinberg equilibrium was evaluated for each variant through Pearson’s chi-square. Genotype frequencies were analyzed by chi square test and the odd ratio (OR) with 95% confidence intervals (CI) between case and control groups. A multivariate logistic regression was used with confounding variables (age, gender and educational level).

For association analysis between genotypes, gene expression and working memory, we used the recessive model considering the minor frequency allele and the risk allele described in various studies [33–37]. The recessive model postulates that the risk of the disease occurs in the homozygotes for risk allele. Therefore, genotypes were divided in two groups for each variant.

Log10^−^ was used to transform the values of cognitive scores and gene expression levels to approximate normal distributions and to conduct an ANCOVA analysis and linear regression adjusted for age, gender, and educational level.

The association between working memory function, variant genetic and expression levels was analyzed by a two-way ANCOVA. The two fixed factors were diagnosis (schizophrenia vs control) and the genotype (recessive model). The expression levels of *NR4A2* were used as a covariant, as well as demographic data including age, sex and education level. The interactions of “diagnosis x genotype”, “genotype x expression” and “diagnosis x genotype x expression” were obtained. Posteriorly, whether a significant effect was identified in any interaction or main effects on the genotype, a simple effects analysis was performed in patients and controls separately. Gender, age and education (self-report of the number of years of study) were used as covariates in the simple effect analysis.

All statistical analyzes were performed using the SPSS Statistics Version 24 software (SPSS Inc., Chicago, IL, USA) and the STATA SE version 12.0 statistical software (Stata Corp, College Station, TX, USA). *p* < 0.05 values were considered statistically significant. For the cognitive tests, we used Bonferroni correction for counteract the problem of multiple comparisons for the five cognitive analyzes.

The calculation of the sample size and the statistical power were performed with the software named G * Power version 3.1.9.6.

## Results

### Participant characteristics

Demographic and clinical characteristics of patients and controls included in the study are shown in Table 1. Gender (*p* = 0.485), education (*p* = 0.295), and age (*p* = 0.066) distributions did not show any statistically significant difference between patients and control volunteers.

**Table 1:** Socio-demographic characteristics, clinical data, and genotype distribution of *NR4A2* in schizophrenia patients and control group

Characteristics	Schizophrenia group (*n* = 187)	Control group (*n* = 227)	*P*
Age, year (±SD)	35.44(±10.07)	38.33 (±13.17)	0.066^a^
Gender, n (%)			
Male	111 (59.4)	127 (55.9)	
Female	76 (40.6)	100 (44.1)	0.485^b^
Education, year (±SD)	10.98 (±3.46)	11.52 (±4.47)	0.295 ^a^
Family history of schizophrenia (%)	64 (34.2)	–	
Age of onset, year (±SD)	23.16 (±7.62)	–	
Disease duration, year (±DS)	12.39(±9.76)	–	
Typical antipsychotics, n (%)	64 (34)		
SSRI n (%)	17 (9)		
**rs34884856 promoter variant**			
Allele n (%)			
2C	211 (56)	261 (57)	0.757 ^b^
3C	163 (44)	193 (43)	
Genotype n (%)			
2C/2C	54 (28.9)	72 (31.7)	
3C/2C	103 (55.1)	117 (51.5)	0.762 ^b^
3C/3C	30 (16.0)	38 (16.7)	
HWE (*p*)	0.14	0.43	
**rs35479735 intron 6 variant**			
Allele n (%)			
2G	168 (45)	211 (46)	0.656 ^b^
3G	206 (55)	243 (54)	
Genotype n (%)			
2G/2G	32 (17.1)	48 (21.1)	
3G/2G	104 (55.6)	115 (50.7)	0.504 ^b^
3G/3G	51 (27.3)	64(28.2)	
HWE (*p*)	0.11	0.89	

Data are presented as mean ± SD. Underlined allele denotes the minor allele

*n* Total participants, *SD* Standard deviation, *SSRI* Selective serotonin reuptake inhibitors, *HWE* Hardy-Weinberg Equilibrium, *3C* Insertion C, *2C* Deletion C, *3G* Insertion G, *2G* Deletion

^a^Mann Whitney *U* test; ^b^Chi-squared test

### Comparison of allele and genotype frequencies between case and control group

The two *NR4A2* genetic variants analyzed in this study have been associated with disorders related to dysfunction of dopaminergic system; their genotypic and allelic frequencies are shown in Table 1. The rs34884856 variant on the promoter (alleles are a cytosine insertion (3C) and a deletion C (2C)), and rs35479735 variant in the intron 6 (a guanine insertion (3G) and a deletion G (2G)), both indels, were analyzed in the group of patients and control participants*.* These two variants were in Hardy-Weinberg equilibrium in both groups of participants. In particular, rs34884856 (3C) minor allele frequency (MAF) was identified in 44 and 43% of patients and control group, respectively for promoter variant. In addition, rs35479735 (2G) MAF was shown in 45% of study cases and in 46% of control individuals for the intron 6 variant. These two polymorphisms were found in strong linkage disequilibrium (D′ = 0.87, *r*^2^ = 0.67).

Distribution of allelic and genotypic frequencies was similar between patients and control volunteers for both variants. The bivariate logistic regression analysis for the different inheritance models was not significantly associated to schizophrenia for either variant (Supplementary 1).

### Comparison of *NR4A2* mRNA peripheral expression between schizophrenia patients and the control group as well as with *NR4A2* genetic variants

The mRNA expression levels of *NR4A2* were not significantly different between the patient and control groups (*p* = 0.766), or between the different genotypes of the two variants in patients or controls (Supplementary 2). Likewise, expression levels showed no differences by sex (*p* = 0.158), drug treatment (*p* = 0.224), or family history (*p* = 0.528) in the schizophrenia group.

### Analysis of the relation of schizophrenia and the rs34884856 promoter variant on cognition

For association analysis between genotypes and working memory, we used the recessive model considering the minor frequency allele and the risk allele described in various studies [33–37]. The recessive model postulates that the risk of the disease occurs in homozygotes for the risk allele. This model is widely used in genetic association studies [57, 58]. Therefore, genotypes were divided in two groups for the rs34884856 promoter variant: 3C/3C homozygous, and 2C carriers (“3C/2C + 2C/2C”), for the rs35479735 intronic 6 variant: 3G/3G homozygous, and 2G carriers (3G/2G + 2G/2G).

The genotypes of the rs35479735 intronic 6 variant (recessive model) did not show significant differences for sociodemographic, cognitive or clinic evaluations in schizophrenia patients and the control group (Supplementary material 3).

Table 2 shows both sociodemographic and clinical variables for recessive model genotypes of the rs34884856 promoter variant. No significant differences were found between genotypes for variables such as age, gender, and education in patients and controls. Furthermore, no significant differences in clinical variables such as age of onset, family history of schizophrenia and treatment between 3C/3C homozygous and carriers of the 2C allele were found in patients.

**Table 2:** Socio-demographic and clinical characteristics of patients and the control group to the rs34884856 promoter variant

	Schizophrenia group			Control group		
	3C/3C *n* = 12	3C/2C + 2C/2C *n* = 41	*p*	3C/3C *n* = 20	3C/2C + 2C/2C *n* = 62	*p*
Age, year (±SD)	32.93 (9.3)	33.50 (10.05)	0.984^a^	38.7 (12.8)	39.94 (11.47)	0.841 ^a^
Gender, n (%) Male	8 (53.3)	26 (68.4)	0.302^b^	10 (50)	30 (48.4)	0.900^c^
Education, year (±SD)	10.58 (2.57)	12.15 (2.97)	0.103^a^	14.05 (3.80)	13.13 (4.38)	0.349 ^a^
Family history of schizophrenia (%)	5 (41.7)	12 (29.3)	0.418^b^			
Age of onset, year (±SD)	20.08 (8.86)	23.35 (6.84)	0.077^a^			
Disease duration, year (±DS)	11.58 (8.27)	10.68 (9.97)	0.515^a^			
Typical antipsychotics, n (%)	5 (33.4)	14 (41.7)	0.284^b^			
SSRI n (%)	1 (8.3)	5 (11.9)	0.718^b^			
**PANSS**						
Positive symptoms	21.00 (7.50)	20.45 (7.98)	0.994^a^			
Negative symptoms	26.58 (9.67)	21.94 (7.19)	0.156^a^			
General symptoms	41.91 (14.82)	37.14 (10.96)	0.323^a^			
PANSS total	88.66 (28.78)	79.33 (22.17)	0.378^a^			
**Working memory test**						
WMI	69.25 (10.59)	75.34 (11.80)	0.103^a^	87.8 (10.04)	80.7 (14.85)	0.086^a^
Arithmetic	4.67 (2.06)	5.63 (2.55)	0.16 ^a^	8.65 (2.30)	7.69 (3.19)	0.32^a^
DS	4.92 (1.24)	6.19 (2.09)	**0.033**^**a**^	6.85 (2.11)	6.39 (1.94)	0.40^a^
BDS task	2.83 (1.11)	4.09 (1.97)	**0.022**^**a**^	4.95 (1.70)	4.5 (1.91)	0.278^a^
LNS	4.67 (2.01)	6.14 (2.36)	0.051^a^	8.50 (1.99)	6.66 (3.08)	**0.027**^**a**^

Data are presented as mean ± SD

This is a subset of the total sample submitted to cognition tasks, which had genotyping and quantification of *NR42* expression levels

*SD* Standard deviation, *n* Total participants, *3C* Insertion C, *2C* Deletion C, *SSRI* Selective serotonin reuptake inhibitors, *PANSS* Positive and negative syndrome scale; recessive model, 3C/3C vs 3C/2C + 2C/2C

^a^Mann-Whitney *U*, ^b^Fisher test, ^c^Chi-squared test

Table 3 shows the ANCOVA analysis conducted for the WMI, working memory subtests (Arithmetic, DS, and Letter-Number Sequencing) and BDS task between patient and control groups according to rs34884856 promoter variant. This analysis showed that performance scores on the BDS task, subtests and WMI were significantly lower in patients compared to healthy controls (Table 3). Further, no genotype effect was identified for performance on any of the subtests or WMI.

**Table 3:** Comparisons among working memory test by diagnostic, genotypic groups and interaction analysis for promoter variant

Test	Diagnosis		Genotype		Diagnosis x genotype		Genotype x expression		Diagnosis x genotype x expression		Statistical Power	Effect size F
	F	*p*	F	*p*	F	*p*	F	*p*	F	*p*		
WMI	**9.44**	0.003*	0.104	0.748	6.11	**0.015**	1.17	0.281	1.32	0.253	0.975	0.906
Arithmetic	**12.257**	0.001*	0.166	0.685	2.59	0.110	1.19	0.276	0.609	0.437	0.947	0.852
DS	**5.26**	**0.024**	2.86	0.093	3.25	0.074	2.57	0.111	0.673	0.413	0.702	0.668
BDS task	**5.01**	**0.027**	**7.48**	0.007*	**6.20**	**0.014**	**7.14**	0.009*	1.719	0.192	0.772	0.706
LNS	4.03	**0.047**	0.028	0.868	**8.89**	0.003^*^	0.796	0.374	2.13	0.147	0.732	0.684

Two-way ANCOVA analysis was performed for each cognitive analysis. The two fixed factors were the genotype (recessive model) and the diagnosis (schizophrenia vs control). The expression levels *NR4A2* was used as a covariant, as well as demographic data including age, sex, and education level. The interaction of diagnosis x genotype, genotype x expression and diagnosis x genotype x expression were obtained

The WMI and its three subtests (DS, Arithmetic, and LNS) that compose it were analyzed. The BDS task that is part of DS subtest was also considered for analysis. This is a subset of the total sample submitted to cognition tasks with 53 case and 82 controls, which had genotyping and quantification of *NR42* expression levels

*WMI* Working memory index, *DS* Total Digit Span, *BDS* Backward Digit Span, *LNS* Letter-Number Sequencing, *F* Test statistic for ANCOVA, *p p*-value

* Bonferroni correction < 0.01

### Working memory and expression levels of the *NR4A2* gene

**Fig. 2:**
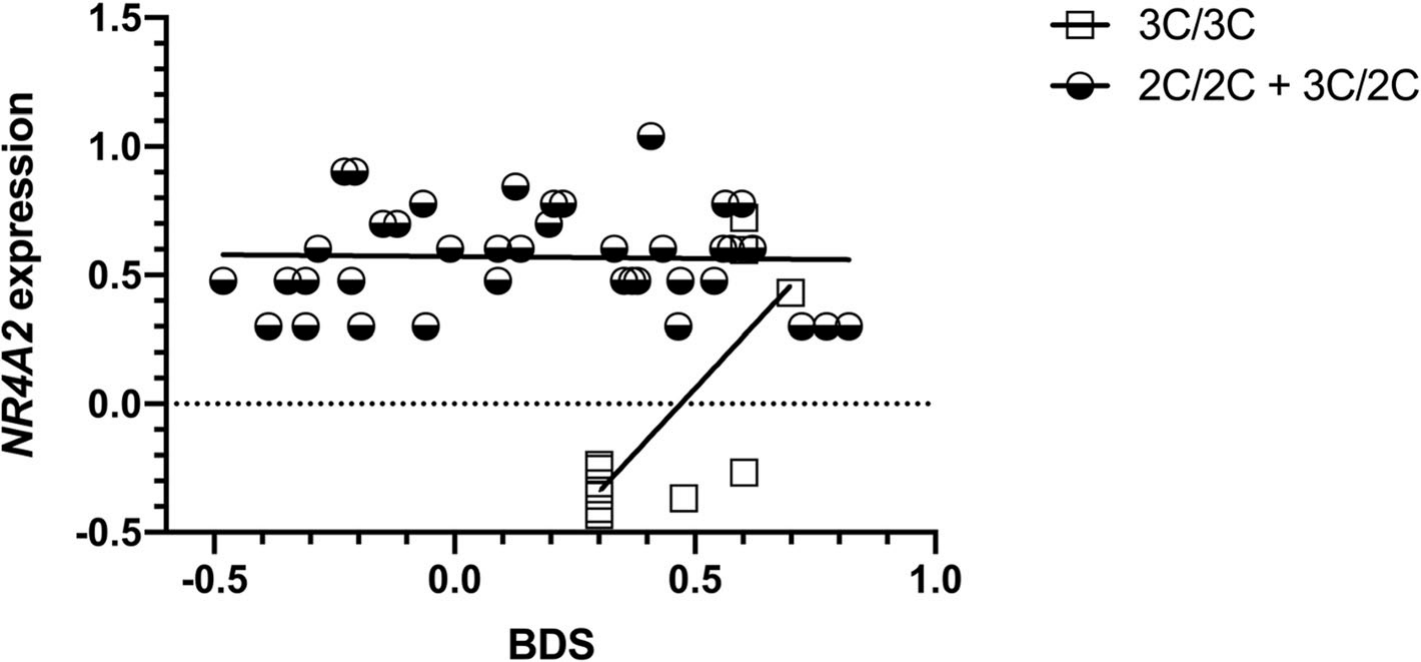
Correlation between Working Memory and *NR4A2* expression levels. Schizophrenia patients homozygous for 3C/3C of rs34884856 promoter variant showed a positive correlation between Backward Digit Span (BDS) and *NR4A2* expression levels. In 3C/3C rs34884856 patients, a decrease of *NR4A2* mRNA expression was related to working memory impairment in schizophrenia

We found a significant interaction effect for diagnosis (schizophrenia vs control) x genotype (recessive model, “3C/3C” vs “3C/2C + 2C/2C”) on WMI (*p* = 0.015), Letter-Number Sequencing (*p* = 0.003) and BDS task (0.014), and a significant genotype x expression effect on the BDS task (*p* = 0.009, Bonferroni correction). Regression lineal analysis showed that BDS test was significantly correlated (simple effect *p* = 0.05, beta = 0.256) with expression levels *NR4A2* in the patients. Also, patients homozygous for the rs34884856 promoter variant (3C/3C) showed a simple effect (*p* = 0.022, beta = 0.76) between expression levels and auditory working memory (BDS) (Fig. 2). This graph shows that only in 3C/3C rs34884856 patients, a decrease of *NR4A2* mRNA expression was related to BDS impairment in schizophrenia.

## Discussion

It is important to mention that this is the first study to analyze the association of two genetic variants of the *NR4A2* gene (rs34884856 in promoter and rs35479735 in intron 6) and *NR4A2* mRNA expression with working memory deficits in schizophrenia.

### Genetic association study between *NR4A2* variants and schizophrenia

This study did not identify any association between the genetic variants analyzed and the schizophrenia diagnosis. Genetic association studies of *NR4A2* variants with this mental disorder in different populations have produced inconclusive and controversial results [26–31, 33, 59]. This could be due to the heterogeneity of the disease diagnosis, the different populations included, and the sample sizes analyzed in different studies. The genotypic frequencies observed in our study are similar to those identified in Asian populations and different from those reported for Caucasian populations in diseases associated with dopamine dysfunction such as schizophrenia [30, 34, 35]. These two variants were found in strong linkage disequelibrium, which agrees with a study in PD of these variants in the Mexican population [36]. However, with the analyzed sample, an association with susceptibility to schizophrenia was not identified for both variables. Although no significant differences were identified by age and sex between cases and controls, other causes such as environmental factors and harmful substances, that were not measured might were influencing the lack of association.

The two genetic variants analyzed in this study have been associated in other populations with different neurological, and psychiatric disorders and addiction, which are diseases with altered dopamine function. It is important to emphasize that the differences between the populations analyzed in other studies, as well as environmental and epigenetics factors, cannot be ruled out, as well as the possibility that NR4A2 is not relevant in the susceptibility to schizophrenia. Accordingly, the study to cognitive endophenotypes in association with variants and levels of expression of the *NR4A2 *gene is important.

### Analysis of *NR4A2* mRNA peripheral expression levels between schizophrenia patients and the control group as well as with *NR4A2* genetic variants

In contrast to other studies [14, 38, 39] where a decreased mRNA expression level of *NR4A2* was identified in brain tissue (DLPFC) of schizophrenia patients, our study showed no significant differences in such expression levels in the mRNA obtained from peripheral blood. It should be noted, however, that the current study analyzed a different type of biological sample (blood vs cerebral cortex tissue). It has been reported that peripheral expression of this gene was found to be significantly decreased in Parkinson’s disease (PD), which is a disease associated mainly with the degeneration of dopaminergic neurons, as well as with aging in different populations [33, 36, 60, 61]. In addition, we did not find an influence of the genetic variants analyzed on the expression levels of the *NR4A2* gene in schizophrenia patients as seen in PD patients [36]. In this neurodegenerative disease it has been previously reported that the levels of expression of the *NR4A2* gene were decreased in 3C homozygous compared to the other genotypes of the rs34884856 promoter variant [36].

### Association between auditory working memory performance and the rs34884856 promoter variant

Endophenotypes are a measurable and supportive tool to improve the ability to identify the association of genetic variants with clinical features, and also are the most direct expression of the effects of genes. In addition, it helps to reduce the heterogeneity of the disease phenotype by facilitating the identification of susceptibility genes. Furthermore, the use of endophenotypes is relevant for a better understanding of the pathophysiology of neuropsychiatric and neurological disorders [9, 62].

Working memory is one of the most widely researched cognitive functions as a cognitive endophenotype of schizophrenia given that it reflects prefrontal cortex alterations [63, 64]. Working memory refers to the mechanisms or processes involved in the control, regulation, and keeping of relevant information active for the execution of complex cognitive tasks [65]. This cognitive function is defined as a system for both temporal storage and manipulation of information, with it participating in key cognitive processes, such as language comprehension, reading, and reasoning [66].

In the present study, an association between the rs34884856 promoter variant of the *NR4A2* gene and auditory working memory in schizophrenia patients was identified. The analysis for the recessive model of this variant (3C/3C versus “3C/2C + 2C/2C”) showed significant differences in BDS; the 3C homozygous patients had a lower scores compared to the 2C allele carriers in this task (Table 2, *p* = 0.022). This is supported by a study that showed the association of genetic variants of this gene with sustained attention in schizophrenia patients in a Caucasian population [32]. Nevertheless, this association was not identified in the control group. However, both the genetic variants analyzed and the cognitive functions evaluated in that previously study are different from those analyzed in our study. It is noteworthy that in our study the relationship between the promoter variant and the working memory functions identified in schizophrenia patients were different from the control group for the BDS task. For the analysis of LNS, the diagnosis * genotype interaction was statistically significant. However, analysis of the single effect in patients and controls separately did not show statistically significant difference.

The difference found between patients and control group regarding the promoter variant can be related to the way in which information is received for processing, and to the affected brain regions in patients. For example, working memory subtests can be classified as verbal and nonverbal. In this context, the left side of DLPFC is more related to verbal tests and the right side of DLPFC is more related to visual spatial tests. Information input divides working memory into auditory (for instance, BDS) and visual spatial. It has been noted that the prefrontal cortex regions involved in simple information storage are different from the regions involved in actively manipulating stored information [67]. In addition, the left hemisphere shows greater pathological alterations in schizophrenia compared to the right hemisphere [68].

Our findings are consistent with studies evaluating other genetic variants that have shown these inconsistencies related to the effects of the allele between different tests and pathological conditions. One of the most widely researched variants demonstrating this effect is the BDNF genetic variant (Val66met) [69, 70]. In this case, the Met allele has a differential effect between patients and controls regarding the same cognitive function.

In addition, the importance of the *NR4A2* gene in relation with metabolism and neuroprotection of dopaminergic neurons allows us to understand the association identified with cognitive endophenotypes of a working memory construct. In order to provide support for the aforementioned, various studies with genes related to dopaminergic metabolism have showed its association with cognitive endophenotypes [71].

### Association between cognitive functions and expression levels of the *NR4A2* gene

This is the first study to identify a positive correlation between *NR4A2* gene expression levels and working memory function in schizophrenia patients. Our results are consistent with previous studies that show that the *NR4A2* gene dosage is significantly related to cognitive function in animal models of schizophrenia [19, 20]. The association of NR4A2 deficiency and reduction in performance on cognitive tasks has been reported for Alzheimer’s disease and attention-deficit hyperactivity disorder animal models [18, 21], as well as with reports of its important role in diverse memory tasks in preclinical studies [17, 22–25]. NR4A2 is also involved in neurodevelopmental disorders and cognitive deficits as reported in clinical trials [15, 16].

We identified a significant positive correlation of *NR4A2* mRNA expression levels with performance on the BDS task in 3C/3C patients (rs34884856 promoter variant). The association between *NR4A2* mRNA expression and better cognitive performance was found in this task of working memory.

In particular, the analysis on the BDS task demonstrated a key association when adjusting variables that affect cognition, such as age, gender, and education level. Our main result was the association of performance on the BDS with the rs34884856 promoter variant and the expression levels of the *NR4A2* gene in schizophrenia patients. For instance, the BDS task allows the evaluation of alterations in auditory working memory, which is related to the left DLPFC.

Our results are consistent with the relationship between cognitive deficits in schizophrenia and dysfunction in different brain regions related to cortex, such as the DLPFC, medial prefrontal cortex, and visual cortex [64]. Therefore, a decrease in dopaminergic neurotransmission in those brain regions could be related to the cognitive deficits in this psychiatric disorder, in particular, the decrease in the *NR4A2* gene in the left DLPFC (area involved in working memory, mainly auditory) in schizophrenia patients.

The first limitations of our study was the sample size for cognitive analysis. In this way, the statistical power identified in the study was borderline for DS, BDS task and LNS (0.72, 0.77, 0.73, respectively), while the effect size was medium for these analyzes. However, no significant differences concerning characteristics that could affect cognitive function were found between the different genotypic groups in either patients or controls. The current research is a pilot study that has shown the relationship of* NR4A2* expression with genotype-dependent working memory. It is important to increase the sample size in future studies in order to verify this initial finding. On the other hand, this study did not analyzed AIMs. Nonetheless, various criteria were used to define the Mexican mestizo [46, 47], and to keep the effects of population stratification to a minimum. In addition, the functional effect of the rs34884856 promoter variant of the *NR4A2* gene has not yet been described. Therefore, this genetic variant could be in linkage disequilibrium with another polymorphism that has a functional effect on the expression levels of this gene and thus, on cognitive function. The NR4A nuclear receptor subfamily has recently been reported to be essential regulator of neurohormonal mechanisms [72]. It could be interesting to evaluate the relationship between the expression of *NR4A2*, the thyroid gland and cognition, since a low thyroid function causes a deterioration in cognition [73], as well as to study other hormones such as aldosterone. Finally, the effect of antipsychotics on cognition is not entirely clear, but there is evidence that the dose, duration, and side effects of antipsychotics may influence on cognitive function of schizophrenia patients [74]. Therefore, another limitation of the present study was that the dose of the antipsychotic treatment used by each patient was not collected, however the type of antipsychotic administered was available, and no significant differences were found between genotypes.

## Conclusion

In conclusion, we identified in this study a significant effect of the relationship of the rs34884856 promoter variant and the expression levels of the *NR4A2* gene on working memory endophenotypes in schizophrenia patients. Our findings suggest that decreased *NR4A2* gene expression could be associated with a deficit in auditory working memory in schizophrenia patients depending on their genotype in a sample from a Mexican population.

## Supplementary Information

**Additional file 1: Supplementary 1.** Allele frequencies and risk calculated using inheritance models. **Supplementary 2.** *NR4A2* gene expression levels in cases and controls according to rs34884856 and rs35479735 variants. **Supplementary 3.** Socio-demographic and clinical characteristics of cases and controls with respect to the rs35479735 intron 6 variant.

## Data Availability

The datasets used and/or analyzed during the current study are available from the corresponding author on reasonable request.

## Abbreviations

AIMs: Ancestral-informative markers
BDS: Backward Digit Span
CIDI: Composite International Diagnostic Interview
CI: Confidence intervals
DLPFC: Dorsolateral prefrontal cortex
DS: Total Digit Span
HRM: High Resolution Melt
MAF: Minor allele frequency
MINI: Mini-International Neuropsychiatric Interview
LNS: Letter-Number Sequencing
OR: Odd ratio
PANSS: Positive and Negative Syndrome Scale
PBMC: Peripheral blood mononuclear cells
qRT-PCR: Real-Time Quantitative Reverse Transcription Polymerase Chain reaction
SNV: Single nucleotide variants
SSRI: Selective serotonin reuptake inhibitors
WAIS-III: Wechsler Adult Intelligence Scale III
WMI: Working memory index
3C: Insertion C
2C: Deletion C
