# Supplementary material for: Working memory deficits in schizophrenia are associated with the rs34884856 variant and expression levels of the NR4A2 gene in a sample Mexican population: a case control study
Source: BMC Psychiatry. 2021 Feb 9;21:86. doi: 10.1186/s12888-021-03081-w (PMC7871565; doi:10.1186/s12888-021-03081-w)
Supplement: Supplementary file 1 — Additional file 1: Supplementary 1. Allele frequencies and risk calculated using inheritance models. Supplementary 2. NR4A2 gene expression levels in cases and controls according to rs34884856 and rs35479735 variants. Supplementary 3. Socio-demographic and clinical characteristics of cases and controls with respect to the rs35479735 intron 6 variant. [file 12888_2021_3081_MOESM1_ESM.docx]

**Working memory deficits in schizophrenia are associated with the rs34884856 variant and expression levels of the *NR4A2* gene in a sample Mexican population: a case control study**

Elizabeth Ruiz-Sánchez^1^, Janet Jiménez-Genchi^2^, Yessica M. Alcántara-Flores^1^, Carlos J. Castañeda-González^3^, Carlos L. Aviña-Cervantes^4^, Petra Yescas^5^, María del Socorro González-Valadez^6^, Nancy Martínez-Rodríguez^7^, Antonio Ríos-Ortiz^2^, Martha González-González^8^, María E. López- Navarro^1^, and Patricia Rojas^1^*

^1^Laboratory of Neurotoxicology, ^4^Department of Psychiatry, ^5^Department of Genetics, ^8^Unit of Cognition and Behavior, Instituto Nacional de Neurología y Neurocirugía, “Manuel Velasco Suárez”, SS, Mexico City, México; ^2^Research Unit, ^3^General Direction, ^6^Health Care Division, Hospital Psiquiátrico Fray Bernardino Álvarez, Mexico City, Mexico; ^7^Epidemiology, Endocrinology & Nutrition Research Unit, Hospital Infantil de México “Federico Gómez”, Mexico City, Mexico.

*Corresponding author:

Patricia Rojas, Ph.D.

Laboratory of Neurotoxicology

Instituto Nacional de Neurología y Neurocirugía. Av. Insurgentes Sur No. 3877, Col. La Fama C.P. 14269, México D.F., México

Tel: (+52 55) 5424 0808; Fax: (+52 55) 5424 0808

e-mail:prcastane@hotmail.com

| **Supplementary 1** Allele frequencies and risk calculated using inheritance models | | | | | | | | |
| --- | --- | --- | --- | --- | --- | --- | --- | --- |
|  |  | | |  |  | | | |
| **Promoter**  **variant**  **rs34884856** | **Allele n(%)**  **2C** | | **3C** | **Models** | | **OR 95%CI *p*** | | |
| Schizophrenia group  (n=187) | | 211 (56) | 163  (44) |  | | |  |  |
|  |  |  |  | Dominant “3C/3C+2C/3C” vs 2C/2C | | | 0.770 (0.470-1.265) | 0.302 |
| Control group  (n=227) | 261 (57) | | 193  (43) | Recessive 3C/3C vs “2C/3C+2C/2C” | | | 0.955 (0.620-1.472) | 0.835 |
|  |  |  |  | Over-dominant 2C/3C vs “3C/3C+2C/2C” | | | 1.220 (0.827-1.800) | 0.315 |
|  |  |  |  | Additive | | | 1.069 (0.805-1.421) | 0.644 |
| **Intron 6**  **rs35479735** | **Allele n(%)**  **2G 3G** | | | **Models** | | | **OR 95%CI** | ***p*** |
| Schizophrenia group  (n=187) | 168 (45) | | 206  (55) |  | | |  |  |
|  |  |  |  | Dominant “3G/3G+2G/3G” vs 2G/2G | | | 0.874 (0.573-1.333) | 0.532 |
|  |  |  |  | Recessive 3G/3G vs “2G/3G+2G/2G” | | | 0.950 (0.563-1.603) | 0.849 |
| Control group  (n=227) | 211 (46) | | 243  (54) | Over-dominant 2G/3G vs “3G/3G+2G/2G” | | | 1.153 (0.782-1.700) | 0.473 |
|  |  |  |  | Additive | | | 1.049 (0.786-1.400) | 0.746 |
| Underlined allele denotes the minor allele. n, total participants; 3C, insertion C; 2C, deletion C; 3G, insertion G; 2G deletion; OR, odd ration; CI, confidence interval; ^a^Chi-squared test. | | | | | | | | |

| **Supplementary 2** *NR4A2* gene expression levels in cases and controls according to rs34884856 and rs35479735 variants | | | | | | | | |
| --- | --- | --- | --- | --- | --- | --- | --- | --- |
|  | Schizophrenia group  (n=112) | |  | | Control group  (n= 118) | *p** | | |
| *NR4A2* mRNA levels | | 1.2 (0.53-1.89) |  | | 1.06 (0.64-2.32) | 0.766^b^ | | |
| Schizophrenia group Control group  (n=112) (n=118) | | | | | | | | |
| **rs34884856 promoter variant**  Genotype | NR4A2 mRNA levels | | | *p* ^Ɨ^ | NR4A2 mRNA levels | *p* ^Ɨ^ | | |
| 2C/2C | 1.23 (0.98-1.64) (n=30) | | |  | 1.00 (0.55-2.5) (n=35) | | |  |
| 3C/2C | 1.21 (0.53-0.96) (n=63) | | | 0.211^a^ | 1.25 (0.81-2.33) (n=54) | | | 0.589 ^a^ |
| 3C/3C | 0.52 (0.42-2.5) (n=19) | | |  | 0.99 (0.71-1.82) (n=29) | | |  |
| Recessive model |  | | |  |  | | |  |
| 3C/3C | 0.52 (0.41-2.52) (n=19) | | | 0.086^b^ | 0.99 (0.70-1.82) (n=29) | | | 0.678 ^b^ |
| 3C/2C+2C/2C | 1.23 (0.61-1.87) (n=93) | | |  | 1.09 (0.60-2.34) (n=89) | | |  |
| **rs35479735**  **intron 6 variant** | | | | | | | | |
| Genotype |  | | |  |  | | |  |
| 2G/2G | 0.77 (0.46-2.50) (n=21) | | |  | 1.10 (0.69-1.84) (n=28) | | |  |
| 3G/2G | 1.20 (0.53-2.02) (n=62) | | | 0.797 ^a^ | 1.19 (0.75-2.34) (n=56) | | | 0.748 ^a^ |
| 3G/3G | 1.23 (0.61-1.64) (n=29) | | |  | 0.99 (0.56-2.55) (n=34) | | |  |
| Recessive model |  | | |  |  | | |  |
| 3G/3G | 1.23 (0.61-1.64) (n=29) | | | 0.841 ^b^ | 0.99 (0.55-2.55) (n=34) | | 0.538 ^b^ | |
| 3G/2G+2G/2G | 1.05 (0.52-2.09) (n=83) | | |  | 1.14 (0.72-2.23) (n=84) | |  |  |
| Data are presents as medians (25 -75 percentile) of mRNA *NR4A2* expression levels; cases and controls were stratified according to a recessive model; n, total participants; 3C, insertion C; 2C, deletion C; 3G, insertion G; 2G deletion; ^a^Kruskal-Wallis test, ^b^Mann-Whitney *U* test. *****Comparison between patients and control group. **^Ɨ^** Comparison between genotype in patients or controls. | | | | | | | | |

| **Supplementary 3** Socio-demographic and clinical characteristics of cases and controls with respect to the rs35479735 intron 6 variant | | | | | | |
| --- | --- | --- | --- | --- | --- | --- |
|  | **Patients** | | | **Control** | | |
|  | 3G/3G  n=15 | 3G/2G+2G/2G n=38 | *p* | 3G/3G  n=21 | 3G/2G+2G/2G n=61 | *p* |
| Age, year (±SD) | 32.93 (9.3) | 33.49 (9.91) | 0.977^a^ | 37.95 (10.62) | 40.21 (12.1) | 0.422^a^ |
| Gender, n (%) |  |  |  |  |  |  |
| Male  Female | 8 (53.3)  7 (46.7) | 27 (69.2)  11 (30.8) | 0.273^c^ | 9 (42.9)  12 (57.1) | 31 (50.8)  30 (49.2) | 0.529^c^ |
| Education,  year (±SD) | 11.4 (2.97) | 11.87 (2.95) | 0.530^a^ | 14.00 (4.21) | 13.14 (4.26) | 0.461^a^ |
| Family history of schizophrenia (%) | 2 (13.3) | 15 (39.5) | 0.066^b^ |  |  |  |
| Age of onset,  year (±SD) | 25.5 (8.88) | 21.44 (6.72) | 0.114^a^ |  |  |  |
| Disease duration,  year (±DS) | 8.33 (10.16) | 11.82 (9.21) | 0.157^a^ |  |  |  |
| **PANSS** |  |  |  |  |  |  |
| Positive symptoms | 21.30 (7.46) | 19.82 (7.75) | 0.530^a^ |  |  |  |
| Negative symptoms | 23.15 (7.74) | 23.08 (8.24) | 0.736^a^ |  |  |  |
| General symptoms | 38.54 (13.46) | 38.25 (11.68) | 0.926^a^ |  |  |  |
| PANSS total | 83.0 (24.78) | 81.17 (24.07) | 0.486^a^ |  |  |  |
| **Working memory test** |  |  |  |  |  |  |
| WMI | 73.07 (12.44) | 74.31 (11.59) | 0.72^a^ | 81.23 (12.7) | 82.85(14.64) | 0.59^a^ |
| Arithmetic | 5.47 (2.06) | 5.39 (2.46) | 0.86^a^ | 7.76 (3.21) | 7.98 (2.97) | 0.90^a^ |
| DS | 4.92 (1.24) | 6.21 (2.07) | 0.65^a^ | 5.95 (1.28) | 6.69 (2.15) | 0.16^a^ |
| BDS task | 4.07 (1.86) | 3.71 (1.90) | 0.46^a^ | 4.38 (1.72) | 4.69 (1.92) | 0.68^a^ |
| LNS | 5.53 (2.56) | 5.92 (2.29) | 0.72^a^ | 7.19 (2.62) | 7.08 (3.07) | 0.93^a^ |
| Data are presented as mean ±SD; SD, standard deviation; n, total participants; ^a^ Mann-Whitney *U*, ^b^Fisher test, ^c^Chi-squared test. This is a subset of the total sample submitted to cognition tasks. | | | | | | |
